# Effects of Long-Term Citrate Treatment in the PC3 Prostate Cancer Cell Line

**DOI:** 10.3390/ijms20112613

**Published:** 2019-05-28

**Authors:** Carmen Caiazza, Massimo D’Agostino, Fabiana Passaro, Deriggio Faicchia, Massimo Mallardo, Simona Paladino, Giovanna Maria Pierantoni, Donatella Tramontano

**Affiliations:** Department of Molecular Medicine and Medical Biotechnology, University of Naples Federico II, 80131 Naples, Italy; carmen.caiazza@unina.it (C.C.); massimo.dagostino@unina.it (M.D.); fabiana.passaro@unina.it (F.P.); d.faicchia@libero.it (D.F.); massimo.mallardo@unina.it (M.M.); spaladin@unina.it (S.P.)

**Keywords:** citrate, prostate cancer, PC3 cells, cell metabolism, organelle homeostasis, autophagy

## Abstract

Acute administration of a high level of extracellular citrate displays an anti-proliferative effect on both in vitro and in vivo models. However, the long-term effect of citrate treatment has not been investigated yet. Here, we address this question in PC3 cells, a prostate-cancer-derived cell line. Acute administration of high levels of extracellular citrate impaired cell adhesion and inhibited the proliferation of PC3 cells, but surviving cells adapted to grow in the chronic presence of 20 mM citrate. Citrate-resistant PC3 cells are significantly less glycolytic than control cells. Moreover, they overexpress short-form, citrate-insensitive phosphofructokinase 1 (PFK1) together with full-length PFK1. In addition, they show traits of mesenchymal-epithelial transition: an increase in E-cadherin and a decrease in vimentin. In comparison with PC3 cells, citrate-resistant cells display morphological changes that involve both microtubule and microfilament organization. This was accompanied by changes in homeostasis and the organization of intracellular organelles. Thus, the mitochondrial network appears fragmented, the Golgi complex is scattered, and the lysosomal compartment is enlarged. Interestingly, citrate-resistant cells produce less total ROS but accumulate more mitochondrial ROS than control cells. Consistently, in citrate-resistant cells, the autophagic pathway is upregulated, possibly sustaining their survival. In conclusion, chronic administration of citrate might select resistant cells, which could jeopardize the benefits of citrate anticancer treatment.

## 1. Introduction

Alterations in metabolism are crucial for supporting aggressive proliferation, evasion from growth-inhibitory signals, cell migration, and metastatic cell dissemination [1].

To survive in a hostile environment, to escape signal control, to migrate and to proliferate, cancer cells undergo “Warburg effect” by reducing oxidative phosphorylation (OXPHOS) and enhancing the rate of glycolysis and lactate production (fermentation), even under aerobic conditions [2]. To warrant a sustained level of glycolysis, cancer cells maintain a low level of citrate for unrestrained PFK1 and PFK2 activity, the latter producing fructose 2,6-bisphosphate (F2,6BP), a powerful allosteric activator of PFK1 [3,4,5]. In addition, citrate serves as an essential precursor for lipogenesis, which in turn is essential for membrane biogenesis. 

Within this picture, the prostate represents a special case. The main function of the prostate gland is to produce large amounts of citrate, secreted thereafter as a component of semen, supplying sperm with the source of energy necessary for their vitality and motility [6]. To this purpose, prostate epithelial cells in the peripheral zone accumulate zinc, which inhibits m-aconitase (ACO2) activity, preventing citrate oxidation. The inhibition of ACO2 and citrate oxidation is lethal in all other mammalian cells, but not for the prostate, which satisfies energy requirements to survive and to sustain citrate production by an increased glycolytic rate. Thus, normal prostate epithelial cells display a high-citrate-producing low-oxidizing phenotype. Interestingly, in prostate cancer cells, a zinc accumulation system does not work [7,8]. Because of the peculiar feature of the normal epithelial prostate cells to evade oxidative phosphorylation at baseline, several findings indicate that prostate cancer (PCa) cells switch to the Warburg effect only in the metastatic stage [6,9]. On the contrary, during early PCa cell transformation, activation of c-MYC fosters a metabolic switch from glycolytic to oxidative metabolism. This allows tumor cells to become progressively more dependent on glutamine metabolism and increases lipid synthesis. As early as 1962, Marberger and co-workers determined that the primary site and metastatic malignancies exhibit decreased citrate levels, and Cooper and Farid in 1963 also identified decreased citrate in PCa [10,11]. In the same line of evidence, Giskeodegard and colleagues reported that the metabolic profile of high grade prostate tumor samples displays decreased concentration of spermine and citrate, as compared with normal tissues, and this feature is associated with tumor aggressiveness [12]. Finally, determination of citrate levels by high-resolution MRI is becoming a key tool in staging prostate cancer [13,14]. At present, androgen deprivation is the main treatment for metastatic prostate cancer, but most patients progress to a more aggressive form of the disease, driven by elevated expression of the androgen receptor. Cancer relapse or recurrence represent major challenges in anti-cancer therapies and could be due to the pre-existence, induction, or selection of drug-resistant cells within the primary tumor.

The well-known ability of citrate to inhibit glycolysis raised the possibility of exploring its anti-oncogenic role. The rationale of this strategy is to counterbalance the Warburg effect by increasing the intracellular concentration of citrate, which could arrest the glycolysis, proliferation, dedifferentiation, and aggressiveness of cancer cells [15,16].

Several reports have shown that the increase in citrate intracellular levels inhibits proliferation, induces apoptosis in cancer cells of different origin, and reduces tumor growth of several xenografts in mice synergizing with anti-cancer treatment [17,18,19,20,21,22,23,24,25,26,27,28,29,30,31,32,33]. The majority of studies investigating the anti-cancer role of citrate describe the inhibitory effect of acute citrate administration, and, according to the literature, the fate of cells surviving citrate treatment has not been investigated yet. 

The aim of the present work was to investigate this issue in PC3 cells. This cell line, originally derived from prostate cancer bone metastasis [34], shares many features of small cell (neuroendocrine) carcinoma (SCNC). In contrast to the majority of prostatic adenocarcinomas that pursue an indolent clinical course, SCNC is highly aggressive. Moreover, although rare as de novo prostate neoplasia, accounting for no more than 1% of all carcinomas of the prostate, SCNCs are often detected as recurrent tumors in patients who have a history of conventional prostatic adenocarcinomas and are subjected to hormonal therapy [35]. In addition, PC3 cells contain negligible levels of intracellular citrate and zinc, even though they possess machineries to uptake both extracellular citrate, through Na^+^- and K^+^-dependent citrate transporters, and the plasma membrane-specific variant of the mitochondrial citrate transporter (pmCiC), and zinc through the zinc transporter ZIP1, albeit at lower levels compared with normal and benign prostate epithelial tissue. On these bases, we consider PC3 cells a suitable model for investigating the effects of chronic citrate treatment [7,36].

The data presented here show that PC3 cells can survive acute citrate treatment, becoming resistant to citrate, and that this feature is only partially reversed by citrate removal.

## 2. Results

### 2.1. Citrate Treatment Affects PC3 Cell Proliferation, Survival, and Metabolism

**Figure 1:**
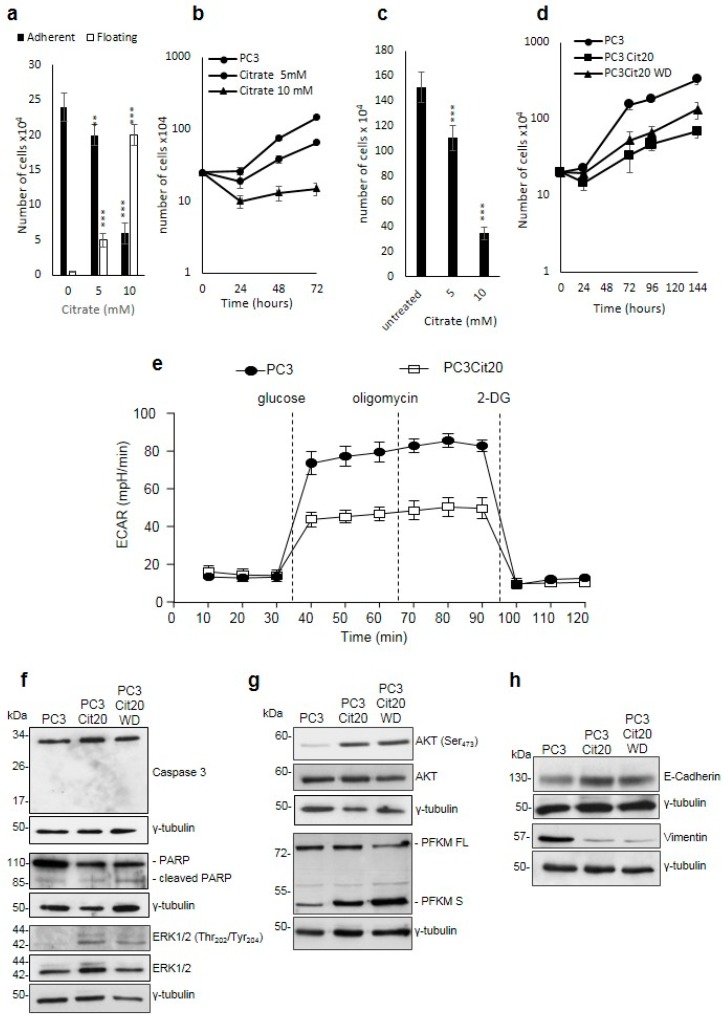
Citrate treatment affects PC3 cell proliferation, survival, and metabolism. (**a**) Citrate impairs the adhesion of PC3 cells in dose-dependent manner. (**b**) Citrate inhibits the proliferation in PC3 cells: 5 and 10 mM citrate was added to a culture medium of PC3 cells at the time of seeding, and cells were counted after 48 h in a Neubauer hemocytometer. Data are expressed as mean ± S.D. of a representative experiment performed in triplicate. The differences between treated and untreated cells are statistically significant (*p* < 0.005 Anova followed by Bonferroni *post*-test). (**c**) PC3 cells were seeded, and citrate was added 24 h after plating. Cell number was determined after 72 h. Data represent the mean of quadruplicate values of two independent dishes. (**d**) Growth curves of PC3 and PC3 Cit20 after citrate withdrawal. Cell number was determined at the indicated time points. Data represent the mean of quadruplicate values of two independent dishes (*p* < 0.001 Anova followed by Bonferroni *post*-test). (**e**) Altered bioenergetic profile in citrate-resistant PC3 cells. Kinetic profile of ECAR in PC3 cells and PC3 cit20 (citrate-resistant) cells. Data are expressed as mean ± S.E.M. of three independent experiments, each of them in triplicate. ECAR was measured in real time, under basal conditions and in response to glucose, oligomycin, and 2-DG. (**f–h**) Lysates from PC3, PC3 Cit20, and PC3 Cit20 WD cells were analyzed by Western blot using the indicated antibodies. γ tubulin was used as loading control. * *p* ≤ 0.05; *** *p* ≤ 0.001, Student *t*-test.

In preliminary experiments, we investigated the effect of acute administration of citrate on the behavior of PC3 cells. To test whether citrate would interfere with cell adhesion, PC3 cells were seeded in the presence of 5 and 10 mM citrate, and the number of adherent and non-adherent cells was counted after 24 h (Figure 1a). The results indicate that citrate impairs cell adhesion in a dose-dependent manner (Figure 1a). Moreover, we evaluated whether citrate, apart from inhibiting adhesion, would influence PC3 cell proliferation. To this aim, cells were seeded in the presence of 5 and 10 mM citrate and were counted after 24, 48, and 72 h. As shown in Figure 1b, citrate also impairs the proliferation of PC3 cells in a dose-dependent manner: indeed, at 72 h, the number of PC3 cells treated with 5 and 10 mM citrate appears lower than the untreated PC3 cells by 66% and 90%, respectively (Figure 1b). To further investigate the possible influence of impaired adhesion on proliferation, we added citrate (5 and 10 mM) 24 h after plating, allowing PC3 cells to adhere to the culture dishes. After 72 h, citrate inhibits proliferation and does not interfere with adhesion (Figure 1c). These results confirm, as reported in other cell systems, that acute citrate treatment induces the inhibition of PC3 cell proliferation. In parallel, we tested the acute effect of citrate administration on EPN cells, a line derived from normal epithelial human prostate spontaneously adapted to grow in culture, and on EPN-PKM cells, which derive from EPN cells transfected with a kinase-negative mutant of the non-receptor kinase Pyk2. We observed that citrate did not affect the adhesion of both EPN and EPN-PKM cells (data not shown). Interestingly, while citrate does not influence the proliferation rate of EPN cells, it slightly inhibits the proliferation of EPN-PKM cells at the concentration of 10 mM (Figure S1a). 

We then decided to test whether PC3 cells would survive to chronic citrate treatment. To this aim, PC3 cells were cultured in the continuous presence of 10 mM citrate, and culture medium was changed three times a week to maintain stable elevated levels of citrate. Surprisingly, a small group of PC3 cells surviving the chronic treatment with citrate started slowly to proliferate (about two weeks). Once this subpopulation of PC3 cells resistant to 10 mM citrate reached confluence (about three weeks), it was harvested and seeded in a standard medium containing 20 mM citrate. We used this higher citrate concentration to be sure to have selected a citrate-resistant subpopulation. In the switch from 10 to 20 mM citrate, few cells did not adhere to the culture dish. Therefore, the entire process to obtain PC3 cells, stably growing in a medium supplemented with 20 mM citrate (hereafter called PC3 Cit20 cells), lasted about three months. PC3 Cit20 cells proliferated at a rate slower than PC3 wild type (doubling time of 66 versus 24 h, respectively) (Figure 1d). To test whether the slow growth rate of PC3 Cit20 cells was strictly dependent on the continuous presence of citrate or rather was a stable feature of these cells, citrate was removed and cells were cultured in a standard medium (hereafter called PC3 Cit20 WD). As shown in Figure 1d, after citrate withdrawal cells maintained a growth rate significantly lower than PC3 cells (*p* < 0.0001), but higher than PC3 Cit20 cells (*p* < 0.0002). In summary, we obtained a subpopulation of PC3 cells stably resistant to chronic treatment with a high concentration of extracellular citrate.

Considering the critical relationship between citrate and glycolysis on the one hand, and glycolysis and aggressiveness of metastatic tumor on the other, we evaluated the glucose metabolism in PC3 and PC3 Cit20 cells. To this aim, the extracellular acidification rate (ECAR), an indicator of glycolysis, was measured using the Seahorse XFe96 Bioanalyzer (Figure 1e). PC3 Cit20 displayed decreased activation of the glycolytic pathway with respect to PC3 cells, as indicated by the reduced level of basal glycolysis and glycolytic capacity (Figure 1e and Figure S1b,c), in agreement with their slow proliferation rate (Figure 1d). 

### 2.2. Citrate Alters Signaling Pathways Governing the Proliferation, Differentiation, and Survival of PC3 Cells

Such observation prompted us to investigate whether changes induced by citrate resistance would affect the expression/activity of some of the principal proteins involved in signaling pathways governing cell survival, proliferation, and differentiation.

Interestingly, PC3 Cit20 cells did not show traits of apoptosis as evidenced by AnnexinV/propidium iodide assays (Figure S2a). In agreement with these results, a lack of Caspase 3 activation and PARP cleavage was observed (Figure 1f). Conversely, citrate induced the activation of the MAPK pathway, as shown by ERK1/2 phosphorylation (Figure 1f). Neither PARP cleavage nor the expression of Caspase 3 or of ERK1/2 was reverted by citrate withdrawal (Figure 1f).

Moreover, citrate induced AKT activation via Ser 473 phosphorylation, which was unaffected by citrate withdrawal (Figure 1g). As the Ser 473 is required for the full activation of AKT, our findings suggest that resistance to citrate might correlate with the full activation of the survival pathway [37].

Because citrate is the main inhibitor of PFK1, we investigated the expression of PFK1 in our cell system. Interestingly, Western blot analysis of the total protein extracts of PC3 Cit20 and PC3 Cit20 WD cells showed that the expression of full-length PFK1 [38] was accompanied by the expression of the shorter form (49 kDa) of PFK1 (Figure 1g). The PFK1 49 kDa form lacks the citrate-binding site, thus rendering the enzyme insensitive to its main allosteric inhibitor. The shorter form, which was barely detectable in PC3 cells, was overexpressed in PC3 Cit20 cells, and its levels remained insensitive to citrate removal. Because the increase in 49 kDa PFK1 parallels that of pAKT, which is described as a key player in the proteolytic process of PFK1 [39], we tested whether the inhibition of AKT could modify the expression of PFK1. Treatment of PC3, PC3 Cit20, and PC3 Cit20 WD with the selective AKT inhibitor Ly294002 (75 µM for 24 h) did not influence the expression of both PFK1 full-length and PFK1 short isoform (Figure S2b).

Finally, citrate resistance induced E-cadherin expression and reduced vimentin expression (Figure 1h), suggesting that PC3 Cit20 cells displayed traits of mesenchymal-epithelial transition, which were by and large unaffected by the removal of citrate. Concerning this latter observation, it is important to note that long-standing ERK1/2 activation, in addition to supporting proliferation, is involved in the regulation of cell differentiation.

### 2.3. Cytoskeleton Dynamics is Altered in Citrate-Resistant PC3 Cells

**Figure 2:**
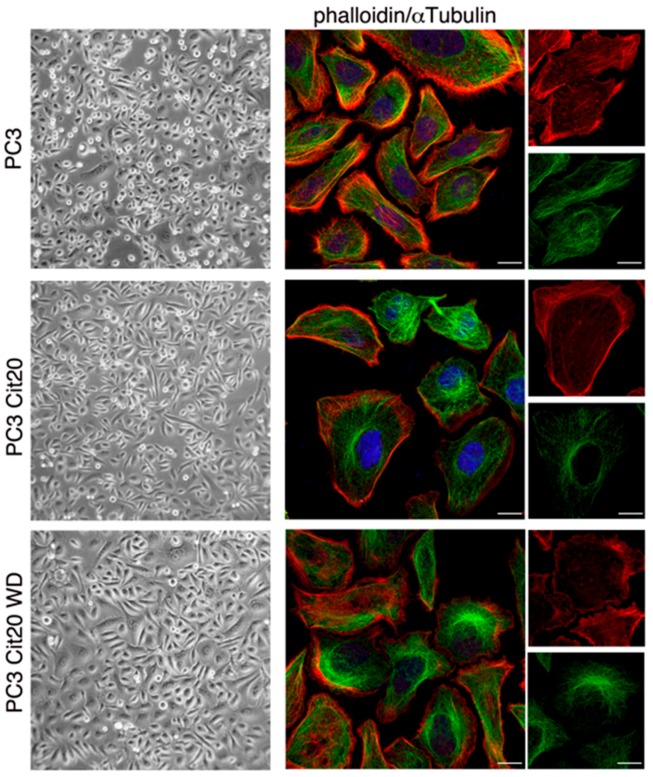
Cytoskeleton dynamics is altered in citrate-resistant PC3 cells. (**left column**) Morphological features of PC3 cells wt, Cit20, and Cit20 WD have been analyzed. Cells were observed with Axiovert 25 (Carl Zeiss, Jena, Germany), and representative pictures were taken with Canon GC5 (Canon Italia S.p.A, 20063 Cernusco sul Naviglio, Milan, Italy) (final magnification 40×). (**right column**) Actin and the microtubule network were labeled using TRITC-conjugated phalloidin, and a specific anti-αtubulin antibody revealed with alexa-488 conjugated secondary antibody, respectively. Nuclei were stained with DRAQ5. Representative images at low (scale bars 10 μm) and higher magnification (rightmost column, scale bars 5 μm) are shown.

PC3 Cit20 cells displayed a morphology that was quite different with respect to PC3 cells with a more extended shape, which largely reverted to a PC3-like morphology upon citrate removal (Figure 2, left column). To obtain deeper insights in the citrate-induced morphological changes, we analyzed actin microfilament and microtubule organization, labeling them with phalloidin and an anti-αtubulin antibody, respectively (Figure 2, right column). PC3 Cit20 cells display a rearrangement of actin cytoskeleton characterized by a significant decrease in filopodia and an increase in large membrane protrusions resembling membrane ruffles (Figure 2, right column). Cortical actin appears comparable in the two cell lines. Moreover, while in PC3 cells the microtubule network is well elongated along the cell axis, it appears less defined at the cell periphery of PC3 Cit20 cells and with a tendency to grow closer to centrosome in the perinuclear region (Figure 2, right column). All these data suggest alteration of cytoskeleton dynamics in PC3 Cit20 cells. Upon citrate withdrawal, the phenotype is partially restored (Figure 2, lower panels).

Interestingly, re-addition of 20 mM citrate to PC3 Cit20 WD induced a rapid morphological reversion, accompanied by a very modest cell detachment, indicating that PC3 Cit20 resistance to citrate is a stable feature of the cells, although they are still responsive to citrate.

### 2.4. The Mitochondrial Network is Altered in Citrate-Resistant PC3 Cells

**Figure 3:**
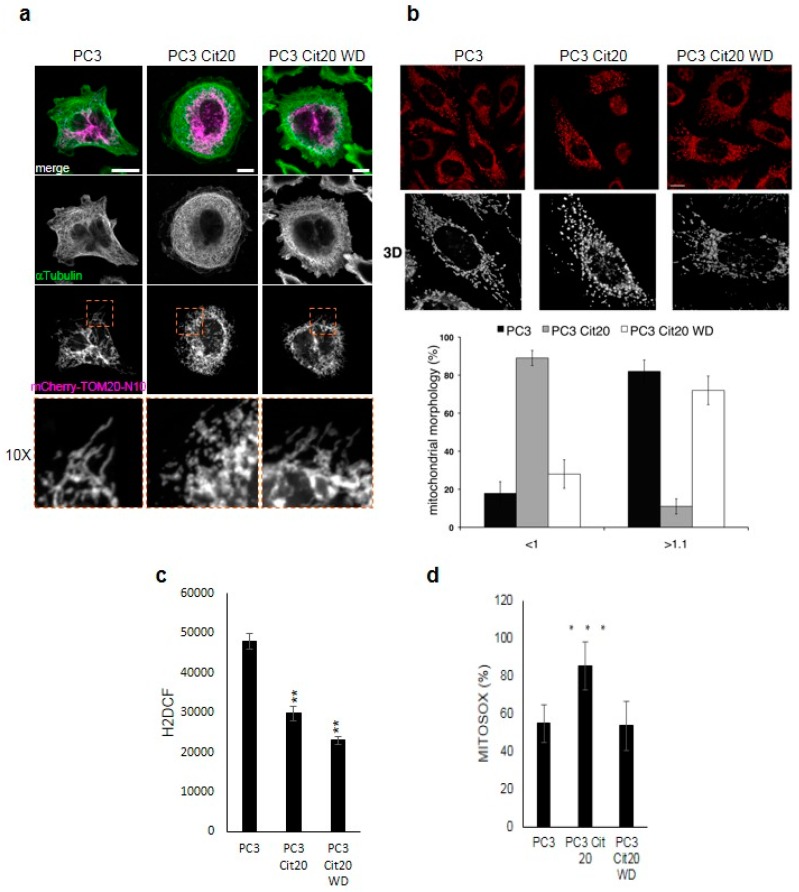
The mitochondrial network is altered in citrate-resistant PC3 cells. Representative images of PC3, PC3 Cit20, and PC3 Cit20 WD cells labeled with mCherry-TOM20-N10 (**a**) or with MitoTracker Red (**b**) are shown. In (a), cells were also stained using a mouse monoclonal antibody against αtubulin. In (b), a single Z-section and 3D reconstruction of a higher magnification region is shown. Quantification of mitochondrial shape according to length (in μm) is shown. Bars, mean ± S.D. Scale bar 10 μm. (**c**) Effects of citrate on ROS production in PC3, PC3 Cit20, and PC3 Cit20 WD cells. ROS levels were estimated indirectly by measuring the fluorescence after 45 min H2DCF addition. Data are expressed as mean ± S.D. of a representative experiment performed in triplicate. (**) indicates statistically significant differences (*p* < 0.05). (**d**) Effects of citrate on mitochondrial ROS production in PC3, PC3 Cit20, and PC3 Cit20 WD cells. ROS levels were estimated by Mitosox. Data are expressed as mean ± S.D. of a representative experiment performed in triplicate. (***) indicates statistically significant differences (*p* < 0.0001).

The different morphology and shape of PC3 Cit20 cells suggest a different organization of the intracellular organelles. Thus, we investigated the morphology and organization of intracellular organelles. The morphology of mitochondria was assessed by overexpressing the fluorescent protein mCherry bearing the N-terminal mitochondrial translocating sequence of TOM20 (mCherry-TOM20-N10) or by using the vital dye Mitotracker Red (Figure 3a,b, respectively). The mitochondrial network of PC3 Cit20 was fragmented, with many small and short mitochondria in comparison with the PC3 cells, which exhibited a branched tubular morphology (Figure 3a,b). The tubular morphology of mitochondria was almost completely restored after 72 h of citrate removal (Figure 3a,b), suggesting that the effect of citrate on mitochondrial morphology was reversible.

ROS production contributes to mitochondrial damage in a range of pathologies and is also important in redox signaling from the organelle to the rest of the cell. To assess whether citrate induced fragmentation could affect mitochondria function, we investigated ROS production in PC3 and PC3 Cit20 cells. As shown in Figure 3c, PC3 Cit20 cells showed a reduced amount of total ROS compared to PC3 cells. Moreover, 72 h citrate withdrawal did not restore ROS production at levels comparable to control cells (Figure 3c). On the other hand, when mitochondrial ROS production was measured by means of MitoSOX Red, an indicator of mitochondrial superoxide, PC3 Cit20 cells produced and/or accumulated a much greater amount of mitochondrial ROS than control cells, which was decreased at a level comparable to those of PC3 upon citrate removal (Figure 3d). We can speculate that the accumulation of mitochondrial ROS observed in PC3 Cit20 cells could be due to the perturbation of mitochondria induced by citrate, which impairs ROS release from the mitochondria.

### 2.5. Exocytic and Endocytic Organelles Are Altered in Citrate-Resistant PC3 Cells

To obtain deeper insight in the intracellular organization of PC3 Cit20, we investigated the morphology and distribution of exocytic and endocytic organelles in citrate-resistant cells in comparison with wild-type cells by confocal microscopy. 

**Figure 4:**
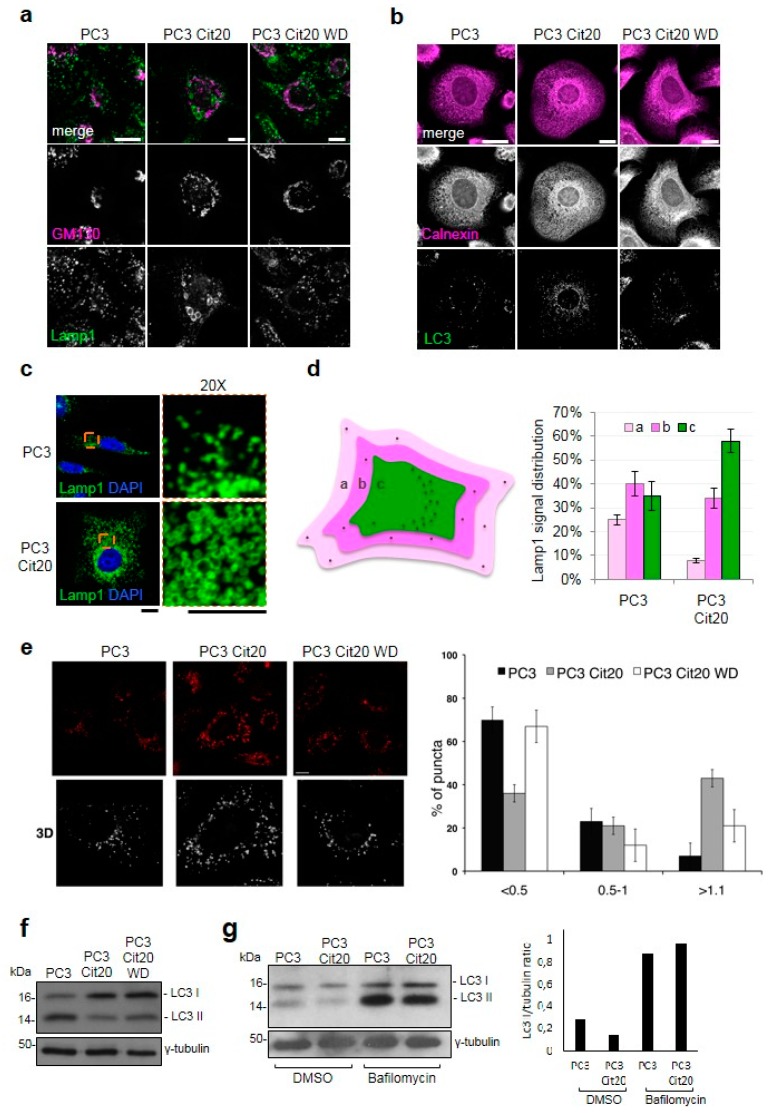
The morphology of Golgi complex and endolysosomal compartment is altered in citrate-resistant PC3 cells. Representative images of PC3, PC3 Cit20, or PC3 Cit20 WD cells labeled with different organelle markers (a-e) are shown. (**a**,**b**) PC3, PC3 Cit20, and PC3 Cit20 WD cells were grown on glass coverslips and subjected to indirect immunofluorescence by using the indicated antibodies to stain Golgi complex (GM130), lysosomal compartment (Lamp1), endoplasmic reticulum (Calnexin), and autophagosomes (LC3). A single focal section is shown. Scale bar: 10 μm. (**c**,**d**) Lysosomal compartment and nuclei of PC3 and PC3 Cit20 cells were labeled by using a mouse monoclonal antibody against Lamp1 and DAPI, respectively. Magnifications (20×) are shown on the right. Scale bar: 10 μm. In (d), lysosome distribution was analyzed as indicated in the scheme and quantified as reported in the histogram on the right. Data are expressed as mean ± S.D. of three independent experiments on a total of 90 cells. (**e**) For each condition, single Z-section (scale bar 10 μm) and 3D reconstruction are shown. Quantification of lysosome size expressed as a percentage of total puncta is shown. Bars: mean ± S.D. (**f**) Western blot analysis of LC3-II protein levels was performed on total cell extracts of PC3, PC3 Cit20, and PC3 Cit20 WD cells. (**g**) As in (f), LC3-II protein levels were analyzed before and after bafilomycin A1 treatment (400 nM) and quantifications are shown in the histogram on the right.

PC3 Cit20 cells showed a defect in the Golgi complex organization, which appeared scattered (Figure 4a), suggesting a putative defect in the secretory pathway. To test this hypothesis, two surface transmembrane proteins, HA-Frizzled4 and GFP-LAT, were exogenously overexpressed and here used as a reporter of the integrity of the secretory pathway. Both HA-Frizzled4 and GFP-LAT successfully reached the plasma membrane of PC3 Cit20 as well as that of the PC3 control cells (Figure S3a,b), indicating that citrate-induced Golgi scattering was not severe enough to compromise protein transport along the secretory route. This observation was further supported by the microscopy analysis of Golgi marker localization, showing that each small dispersed Golgi stack was properly labeled with GM130 and Golgin-97, markers of cis- and trans-cisternae of Golgi, respectively (Figure S4a). Thus, in this condition, a Golgi ribbon was only partially disassembled and separated Golgi stacks maintained their structural and functional features.

Strikingly, the lysosomal compartment of PC3 Cit20 cells was enlarged in comparison with PC3 control cells (Figure 4a). Indeed, as revealed by the lysosome associated membrane protein 1 (Lamp1) antibody or by Lysotracker dye, the lysosomes increased in number (about twofold more) and in size (Figure 4a,c,e). Interestingly, these alterations were restored in the major part of the cells upon citrate withdrawal (Figure 4a,e). Moreover, Lamp1-positive structures were also differently localized within the cells, and most of them appeared to concentrate near the microtubule-organizing center (MTOC) (Figure 4c,d). Interestingly, when these structures were relocalized to the cell periphery by overexpressing the GTPase Arl8b-GFP involved in kinesin-dependent transport towards the cell periphery [40], the Golgi complex returned to assume its physiological compact morphology (Figure S4b, upper panels). This evidence suggests that Golgi fragmentation could be due to a steric hindrance effect caused by the accumulation of enlarged lysosomes to the MTOC. This hypothesis was also consistent with the effect of GFP-RILP, a GTPase controlling the dynein-dependent transport towards MTOC [40], whose overexpression in PC3 cells caused the centripetal localization of Lamp1-positive structures to the MTOC and a significant Golgi fragmentation phenotype (Figure S3b, lower panels). Such alterations of the lysosomal compartment could also reflect a different degradative behavior of the cells, which was confirmed by the more numerous LC3 positive dots found in the PC3 Cit20 compared to the PC3 cells (Figure 4b). Surprisingly, in basal conditions, we found that LC3-II, the form of LC3 recruited on autophagosome membranes, was significantly reduced in PC3 Cit20 with respect to PC3 cells and was unaffected by citrate withdrawal (Figure 4f). On the other hand, LC3-II was increased upon bafilomycin treatment in both PC3 and PC3 Cit20 cells (Figure 4g), suggesting that in PC3 Cit20 cells the autophagic flux is more rapid.

## 3. Discussion

Several studies have reported that elevated levels of citrate inhibit proliferation and induce the cell death of multiple cultured cancer cells such as those of ovarian, mesothelioma, pancreas, lung, gastric, melanoma, and prostate cancers) [17,18,19,20,21,22,23,25,26,27,28,29,30,31,32,33]. Elevated levels of intracellular citrate could be achieved either by ATP-citrate lyase inhibition [41,42] and/or by administration of extracellular citrate, which may be taken up through plasma membrane citrate transporter(s) [36,43].

Citrate inhibits proliferation via inhibition of the IGF-1R/PI3K/AKT axis, induces activation of the PTEN-eIF2a pathway in lung tumor cells [25], inhibits Mcl-1 [18], promotes apoptosis via the activation of Caspase 2 or 8 in pleural mesothelioma and gastric cells [21,22], and finally induces autophagy in ovarian cells [20,21], all findings indicating that citrate might affect different pathways according to the cell context. In addition, citrate increases the sensitivity of tumor cells to anticancer treatment [17,18,19,20,21,22,23,24,25,26,27,28,29,30,31,32,33]. Furthermore, the loss of citrate synthase results in markedly unregulated glycolysis, decreased citrate production, and accelerated tumor malignancy [30]. Decreased blood citrate levels have been associated with certain tumors, including those of the lung, bladder, and pancreas [44]. Moreover, citrate concentration in human seminal fluid from prostate cancer patients is 2.7-fold lower than normal patients [45]. All the above information, together with the diagnostic use of citrate level determination in the staging of prostate cancer, is the foundation of the present work.

Our results show that PC3 cells surviving short-term treatment with a high concentration of extracellular citrate (10 and 20 mM) become resistant to chronic citrate administration. PC3 Cit20 cells grow at a slower rate than PC3 cells, with a doubling time of about 72 h. This feature was apparently specific of PC3 Cit20 since citrate withdrawal did not restore a faster growth rate. Conversely, the morphology of PC3 Cit20 cells, which is substantially different from that of PC3 cells, partially reverted to a PC3-like morphology upon citrate withdrawal. 

As described by Franklin and coworkers, PC3 cells have lost the ability to concentrate citrate, and the authors suggested that this feature of PC3 cells may induce metabolic changes that accompany progression towards a more aggressive and undifferentiated phenotype [46]. Citrate resistance profoundly influences the metabolic profile of PC3 Cit20 with respect to PC3 cells. Thus, PC3 Cit20 cells possess levels of glycolysis significantly lower than those of PC3 cells. This change would apparently suggest that PC3 Cit20 cells undergo a metabolic rewiring towards a phenotype less aggressive than the PC3 counterpart [47,48,49]. Indeed, while citrate has been reported to induce cell death in several cell lines via activation of the apoptotic pathway, PC3 Cit20 cells do not show signs of apoptosis, as demonstrated by the lack of Caspase 3 and PARP cleavage. Moreover, PC3 Cit20 cells show traits of mesenchymal-epithelial transition, as indicated by the increase in E-cadherin and the decrease in vimentin expression, which were not reverted by citrate removal. Similar changes have been observed by Ren and colleagues in A549 lung cancer cells and are in line with the evidence linking low levels of citrate with de-differentiation [23,25].

Importantly, PC3 Cit20 cells display a sustained ERK1/2 phosphorylation, which was not changed by citrate withdrawal. Apart from regulating cell proliferation, sustained ERK1/2 activation plays a pivotal role in cellular differentiation [50]. Although slow-growth, reduced glycolysis and differentiation would support all together the image of PC3 Cit20 cells as a less aggressive phenotype, the lack of any sign of apoptosis, i.e. resistance to cell death, is a hallmark of highly malignant cancer cells. 

The increase in the shorter PFK1 form supports this hypothesis. This observation is interesting in a prostate context considering that PSA belongs to the Kallikrein family, a group of serine proteases able to cleave native PFK1 enzymes to form shorter, citrate-insensitive PFK1 fragments [38,51,52]. The cleavage of the C-terminal segments of the holoenzyme, known to stabilize the tetrameric quaternary structure of the native eukaryotic protein, makes the holoenzyme more susceptible to dissociation [53]. As the distal portion of the C-terminus is responsible for the formation of tetrameric holoenzymes, it seems very likely that the shorter fragments, lacking a major part of C-terminus, can assemble only in dimeric forms. It has been reported that the PI3K/Akt/mTOR signaling pathway plays a crucial role in the proteolytic processing of PFK1, from 85 kDa to 49 kDa fragments, in breast and prostate cancer cell lines [54,55]. Thus, activated AKT in the PC3 Cit20 cells may suggest the involvement of this pathway in the proteolytic processing of PFK1 in these cells. The presence of trace amounts of PFK1 in PC3 cells is intriguing and raises the possibility that, within PC3 cells, there could be a sub-population bearing by default the shorter form of PKF1, which would give a Darwinian advantage under the pressure of citrate selection. Hence, the lack of effect upon citrate removal on the expression of PFK1 49 kDa is not surprising. However, the presence of the shorter PKF1 does not explain it all. However, PC3 Cit20 cells are still responsive to citrate, as demonstrated by the observation that some citrate effects are at least in part reversible. 

We show here that the mitochondrial network of PC3 Cit20 cells is fragmented with many small and short mitochondria in comparison with the PC3 cells, which exhibited a branched tubular morphology. Mitochondrial fragmentation is reverted upon citrate removal. Excessive mitochondrial fragmentation has been associated with cell death in pancreatic PANC-1 cells, as well as colorectal, gastric, and breast cancer cells, and Drp1-mediated mitochondrial fission suppresses breast cancer cell invasion [56]. A reciprocal link between mitochondrial morphology and ROS formation is a critical issue for mitochondrial function, as mitochondrial shape and structure are intimately linked to the control of redox homeostasis by modulating ROS as a downstream signal [57]. Consistent with their glycolytic phenotype, PC3 cells show total levels of ROS that are higher than those of PC3 Cit20 cells, and the withdrawal of citrate does not revert the total citrate-induced ROS decrease. Conversely, PC3 Cit20 cells display a high mitochondrial ROS production/accumulation that was reverted by citrate removal. We cannot exclude the possibility that the high levels of mitochondrial ROS displayed by these cells represent a sign of ROS accumulation rather than ROS over-production. Thus, it is conceivable that the citrate-induced fragmentation of mitochondria interferes in some way with the opening of the mitochondrial permeability transition pore (mPTP), paradoxically protecting PC3 Cit20 cells from the ROS overflow, thus inhibiting apoptotic cell death [58]. A similar case has also been observed in several HCC cell lines, where the fragmented mitochondrial phenotype induced by the overexpression of dynamin-related protein 1 (Drp1) or knockdown of mitofusin 1 (Mfn1) was associated with ROS overproduction, AKT activation, the evasion of apoptosis, and the induction of general autophagic pathway [59].

Autophagy is an important catabolic process that delivers cytoplasmic material to the lysosomes for degradation. Autophagy promotes cell survival by the elimination of damaged organelles and protein aggregates as well as by facilitating bioenergetic homeostasis [60]. In our cell system, PC3 Cit20, resistance to citrate increased ROS production and accumulation within mitochondria, thereby causing organelle damage that, in turn, should be expected to promote cell death. However, as shown before, PC3 Cit20 cells do not show sign of apoptosis; rather, survival and proliferation pathways are activated. In this picture, the autophagic pathway could counterbalance the accumulation of damaged organelles promoting their degradation. Consistent with this, we found that, in PC3 Cit20 cells, the autophagic pathway is upregulated, thereby promoting the removal of damaged mitochondria and cell survival. Moreover, as expected in the case of a more rapid autophagosomal maturation process, the lysosomal compartment appeared larger and relocalized to the microtubule-organizing center (MTOC), reflecting the more sustained degradative activity of the cells in this particular growth condition.

Overall, our data indicate that, under the selective pressure of a hostile microenvironment, PC3 cells were forced to undergo metabolic reprogramming modifying their metabolic features in order to survive. Among acutely treated cells, a sub-population could survive and adapt to chronic citrate treatment and become citrate-resistant. This feature of PC3 Cit20 is accompanied by diverse and contrasting behaviors: on one hand, PC3 Cit20 cells show a slow-growing, more differentiated, less glycolytic, and thus potentially less aggressive phenotype. On the other hand, these cells appear more malignant because they do not undergo apoptosis, in spite of mitochondrial fragmentation and possibly because of the impairment of mitochondrial ROS release, and they also use autophagy to survive. Moreover, some of the citrate effects can be reverted, although not completely. Thus, PC3 Cit20 cells appear to possess a very plastic phenotype, able to adapt to “all seasons,” and for this reason are potentially very dangerous. We do not know whether a similar phenotype is present in human cancer specimens and/or whether cells within the tumour can undergo modifications comparable to the citrate-resistant phenotype. However, if so, citrate-resistant cells could jeopardize the promising citrate anti-cancer strategy. 

## 4. Materials and Methods

### 4.1. Chemicals 

Chemicals were purchased from the following manufacturers: Dulbecco Modified Eagle’s Medium/HAM F12 from Gibco Cell Culture, Thermo Fisher Scientific—Invitrogen, (Carlsbad, CA, USA); penicillin, streptomycin, fetal calf serum (FCS), bovine serum albumin (BSA), and phosphate-buffered saline (PBS) from Eurobio (Les Ullis Cedex, France); sodium citrate dihydrate #3646-01 from J.T.Baker—Fisher Scientific Italia (Rodano, Italy); ECL System from Amersham Pharmacia (Buckinghamshire, UK), Bio-Rad assay and prestained Bio-Rad (München, Germany); protease inhibitor cocktail tablets from Roche Diagnostics (Meylan, France); 2’-7’-dichlorodihydrofluorescein diacetate (H2DCFDA) (#D399), MitoSOX™ Red mitochondrial superoxide indicator (#M36008), MitoTracker Red CMXRos (#M7512), Lysostracker red DND-99 (#L7528), DRAQ5 (#62254), and Ly 294002 (#PHZ1144) from Thermo Fisher Scientific—Invitrogen, (Carlsbad, CA, USA). 

### 4.2. Antibodies

The following antibodies were purchased: anti-phospho-AKT (S473) D9E #4060, anti-AKT #9272, anti-Caspase-3 #9662, anti-LC3 A/B (CST#4108), anti-vimentin #5741, anti-GM130 #12480, anti-phospho p44/42 MAPK (T202/Y204) #4370s, anti-LC3 #4108 (used for western blotting) from Cell Signaling Technology (Leiden, The Netherlands); anti-E-cadherin #610182 from BD Biosciences (San Jose, CA, USA); anti-calnexin #SPC-108 from Stress Marq Biosciences Inc. (Victoria, Canada); anti-CD107a/Lamp1 (clone H4A3) #SAB4700416, anti-αTubulin (clone B512) #T5168, anti-γtubulin #T6657 from Sigma-Aldrich (St. Louis, MI, USA); anti-GOLGIN-97 #A-21270 Thermo Fisher Scientific—Invitrogen, (Carlsbad, CA, USA); anti-LC3 (clone 5F10) #0231, used for immunofluorescence, from Nanotools (Teningen, Germany); anti-PFKM #a5477 from Abclonal Technology (Woburn, MA, USA); anti-PARP #sc-7150 from Santa Cruz Biotechnology, Inc (Santa Crus, CA, USA). Alexa-Fluor (488 and 546) secondary antibodies A11029, A11030, A11034, and A11035, were from Thermo Fisher Scientific—Invitrogen, (Carlsbad, CA, USA) and horseradish peroxidase (HRP)-conjugated secondary antibodies used for Western blot analyses were from Amersham Pharmacia (Buckinghamshire, UK).

### 4.3. Plasmids and Cell Transfections

The plasmids were provided as follows: GFP-RILP was kindly provided by Cecilia Bucci, University of Salento, Lecce, Italy; Arl8b-GFP (ADDGENE #67404) by Richard Kahn Lab (Emory University Department of Biochemistry, Atlanta); mCherry-TOM20-N10 (ADDGENE #55146) was provided by Michael Davidson Lab (Florida State University, Department of National High Magnetic Field Laboratory, Tallahassee). 

Plasmid transfections were performed as previously reported [61] by using X-tremeGENE HP DNA Transfection (Roche Italia, Monza MB, Italy), according to the manufacturer’s protocol. 

### 4.4. Cell Culture and Growth Curve

PC3 cells were kindly provided by Dr. Hayden G. Coon, Senior Investigator, Laboratory of Cell Biology, NCI-NIH, Bethesda, Maryland, USA, in 1985. EPN cells, spontaneously immortalized, prostate epithelial cells derived from human normal prostate tissue were obtained in our laboratory. The EPN-PKM3 cell line was obtained as previously described by transfection of EPN cells with plasmids bearing PKM, a kinase-negative mutant for Pyk2 constructed by replacing Lys 475 with Ala residue and pCMVneo containing the fragment of gene phosphotransferase neomycin. All cells were routinely cultured in DMEM/F12 supplemented with 3% FCS (standard medium) [62]. For proliferation assays, cells were seeded in 60 mm culture dishes in a standard medium or in the presence of citrate (5, 10, and 20 mM according to the experimental scheme). Triplicate dishes per experimental time point were trypsinized, and cell number was determined by counting cell suspension in a Neubauer hemocytometer. The values reported represent the mean ± S.D. of at least three independent samples per experimental point. 

For isolation of the citrate-resistant cells, PC3 cells were seeded at a concentration of 3 × 10^6^ cell/100 mm dishes in a standard medium and 10 mM citrate and medium was replaced three times a week. When cells reached confluence, they were trypsinized and seeded in a standard medium containing 20 mM citrate. After a wave of modest detachment, PC3 Cit20 cells stabilized. The latter procedure was repeated three times with the same results. 

### 4.5. Determination of Total ROS and Mitochondrial ROS Production

Fluorimetric determination of intracellular ROS was performed by using 2’-7’-dichlorodihydrofluorescein diacetate (H2DCFDA) as already reported [63]. Briefly, PC3, PC3 Cit20, and PC3 Cit20 WD (72 hours citrate withdrawal) were trypsinized, and 10^6^ cells were re-suspended in 1 mL of HBSS containing H2DCFDA (25 µM) and incubated at 37 °C in a CO_2_ incubator. After 45 min, cells were washed twice with HBBS and re-suspended in 1 mL of HBSS, and 100 µL of cell suspension was seeded in 96-well plates. H2DCFDA was measure in a plate reader, Synergy HT, BIOTEK (Winooski, VT 05404, USA). Experiments were performed at least three times, with six replicates for each experimental point.

For the detection of mitochondrial ROS, cells were harvested and washed once with HBSS. A total of 1 × 10^6^ cells were suspended in 1 mL of HBSS containing MitoSOX (5 μM) and, protected from light, incubated for 10 min. Cells were then washed gently twice with HBSS and re-suspended in 200 µL of warm HBSS for flow cytometry detection. Experiments were performed at least three times with three replicates for each experimental point.

### 4.6. Measurements of ECAR

Real-time measurements of the extracellular acidification rate (ECAR) were measured using an XFe-96 Extracellular Flux Analyzer (Seahorse Bioscience) [64]. Cells were counted using an automated cell counter (Countess from Life Technologies), seeded in XFe-96 plates (Seahorse Bioscience) at a density of 2 × 10^4^ cells/well and incubated overnight at 37 °C in a 5% CO_2_ atmosphere. Cells were counted before and after experiments to ensure the same number of cells. ECAR was measured in XF media in a basal condition and in response to 10 mM glucose, 4 μM oligomycin, and 100 mM of 2-deoxy-d-glucose (2-DG). Basal glycolysis was calculated after glucose injection (subtracting the ECAR rate inhibited by 2DG), glycolytic capacity is the difference between oligomycin-induced ECAR and 2-DG-induced ECAR. Experiments with the Seahorse system were done with the following assay conditions: 3 min mixture, 3 min wait, and 3 min measurement. Data are expressed as mean and S.E.M. from at least two independent experiments performed in triplicate for each condition. 

### 4.7. Annexin V/PI Apoptotic Assay

PC3, PC3 Cit20, PC3 WD were harvested, washed with PBS, and resuspended in binding buffer (eBioscience Thermo Fisher Scientific, Carlsbad, CA, USA) at a concentration of 1 × 10^6^ cells/mL. Cells were stained with Annexin-V-FITC (Immunotools, Friesoythe; Germany) and propidium iodide (PI) for 15 min at room temperature, in the dark. Data were acquired on a BD Accuri C6 flow cytometer (BD Biosciences, San Jose, CA, USA) and analyzed using BD Accuri C6 software.

### 4.8. Immunoblotting

Cells lysis was performed in JS lysis buffer (50 mM HEPES, 150 mM NaCl, 1 mM EDTA, 1 mM EGTA, 10% glycerol, and 1% Triton-X-100) supplemented with protease and phosphatase inhibitors. Protein concentration was estimated by a Bradford assay, and 30 μg/lane of total proteins were separated on SDS gels and transferred to nitrocellulose membranes. Membranes were treated with a blocking solution (25 mM Tris, pH 7.4, 200 mM NaCl, 0.5%, 0.025% TWEEN20) containing 5% non-fat powdered milk and incubated overnight with primary antibodies. Membranes were incubated with an HRP-conjugated secondary antibody, and chemiluminescence was determined using the ECL detection system. Densitometric analysis was performed using the NIH Image software (Bethesda, MD, USA). 

### 4.9. Bafilomycin Treatment

Bafilomycin A1 (#S1413, Selleckchem, Houston, TX, USA) was dissolved as a 0.4 mM stock in dimethyl sulfoxide (DMSO) and stored in aliquots at −20 °C. After 4 h of treatment with 400 nM Bafilomycin A1, cells were washed three times with PBS before protein extraction. During the experiments, control cells were incubated with the same final concentration of DMSO (0.1%).

### 4.10. Fluorescence Microscopy 

Immunofluorescence staining was performed as previously reported [65,66]. Briefly, cells grown on glass coverslips were washed with PBS and fixed in 3.7% formaldehyde at room temperature for 30 min or 100% methanol at −20 °C for 5 min. After fixation, cells were washed with PBS and permeabilized by incubation in blocking buffer (PBS containing 1% BSA, 0.01% Sodium Azide and 0.02% Saponin) for 10 min at room temperature. Cells were then incubated with the indicated primary antibodies diluted in the same blocking buffer for 1 h at room temperature. Cells were washed three times with PBS and incubated with the corresponding secondary antibodies for 35 min at room temperature. Finally, coverslips were washed in distilled water and mounted onto glass slides with the Prolong Gold anti-fade reagent with Dapi (#P36935, Thermo Fisher Scientific—Invitrogen, Carlsbad, CA, USA).

For mitochondria labeling, cells cultured on 12-mm-diameter glass coverslips were incubated with 200 nM MitoTracker^®^ Red CMXRos (Molecular Probes) for 20 min in culture medium as previously described [67]. After incubation, cells were fixed with cold methanol for 5 min, washed with PBS and then mounted in 50% glycerol in PBS. 

Lysotracker was used to label lysosomes as previously described [68,69,70]. Briefly, cells grown on coverslips were incubated with Lysotracker probe (Molecular Probes) for 1 h at 37 °C before fixation. 

Images were collected using a laser-scanning microscope (LSM 510 or 700, Carl Zeiss Microimaging, Inc. Oberkochen, Germany) equipped with a planapo 63× oil-immersion (NA 1.4) objective lens by using the appropriate laser lines. Z-slices from the top to the bottom of the cell were collected, and 3D reconstruction was carried out using LSM 510 software [69]. The number and size of fluorescent structures were determined with ImageJ software [67,69]. Lysosome distribution was quantified using Fiji. Individual cells were outlined as Region of Interest (ROI) using a cytosolic protein marker, and whole cell Lamp1 signal fluorescence was measured. ROI was then decreased by 20%, and Lamp1 signal fluorescence was measured at each decrease. To generate a Lamp1 distribution curve, the signal intensity of each fraction was divided by the total cell signal. Twenty cells were quantified per cell treatment and averaged to quantify the population of lysosome distribution.

### 4.11. Statistical Analysis 

Where applicable, data are expressed as mean ± S.D. Statistical analysis was performed by analysis of variance followed by a Bonferroni post-test or by a Student’s *t*-test. *p*-Values <0.05 were considered statistically significant.

## Supplementary Materials

Supplementary materials are available online at https://www.mdpi.com/1422-0067/20/11/2613/s1.

## Abbreviations

ACO2	m-aconitase
Cit	citrate
F2,6BP	fructose 2,6-bisphosphate
PCa	prostate cancer
PFK1	phosphofructokinase 1
