# Supplementary material for: Effects of Long-Term Citrate Treatment in the PC3 Prostate Cancer Cell Line
Source: Int J Mol Sci. 2019 May 28;20(11):2613. doi: 10.3390/ijms20112613 (PMC6600328; doi:10.3390/ijms20112613)
Supplement: Supplementary file 1 [file ijms-20-02613-s001.pdf]

Figure S1

a

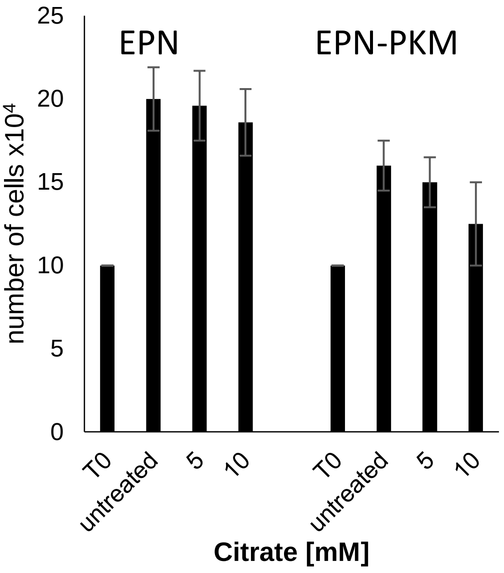

b

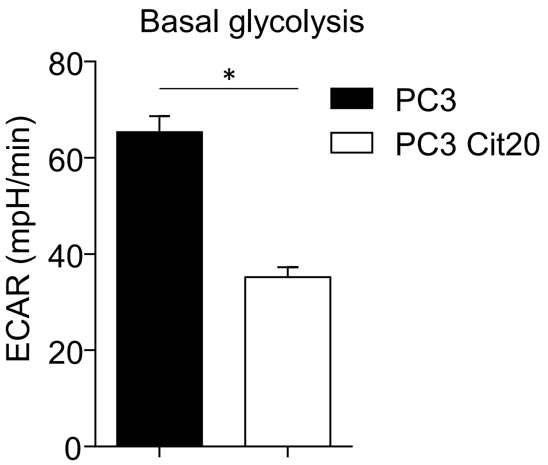

c

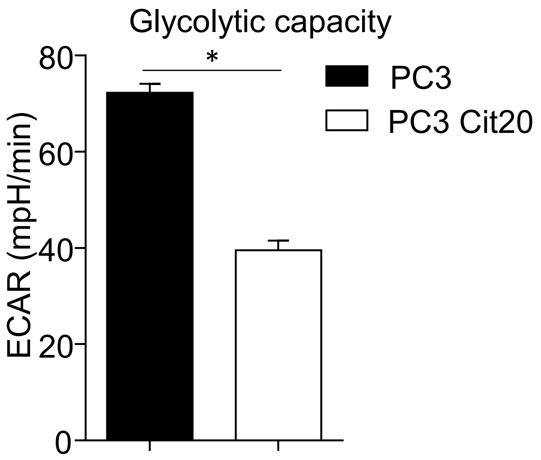

Figure S1. Analysis of citrate treatment on proliferation of EPN and EPN-PKM cells and on the metabolism of PC3 and PC3 Cit20 cells.
(a) Citrate 5 mM and 10 mM was added to culture medium of EPN and EPN-PKM cells at time of seeding and cell were counted after 48 hrs. Cell number was determined in a Neubauer hemocytometer. Data are mean ±SD of a representative experiment performed in triplicate. Note that citrate slightly inhibits the proliferation of EPN-PKM cells, but not of EPN cells.
(**b and c**) **Basal glycolysis and glycolytic capacity in PC3 cells and PC3 Cit20 cells.** Data are expressed as mean ± S.E.M. of three measurements, each of them in triplicates, deriving from three independent experiments.

Figure S2

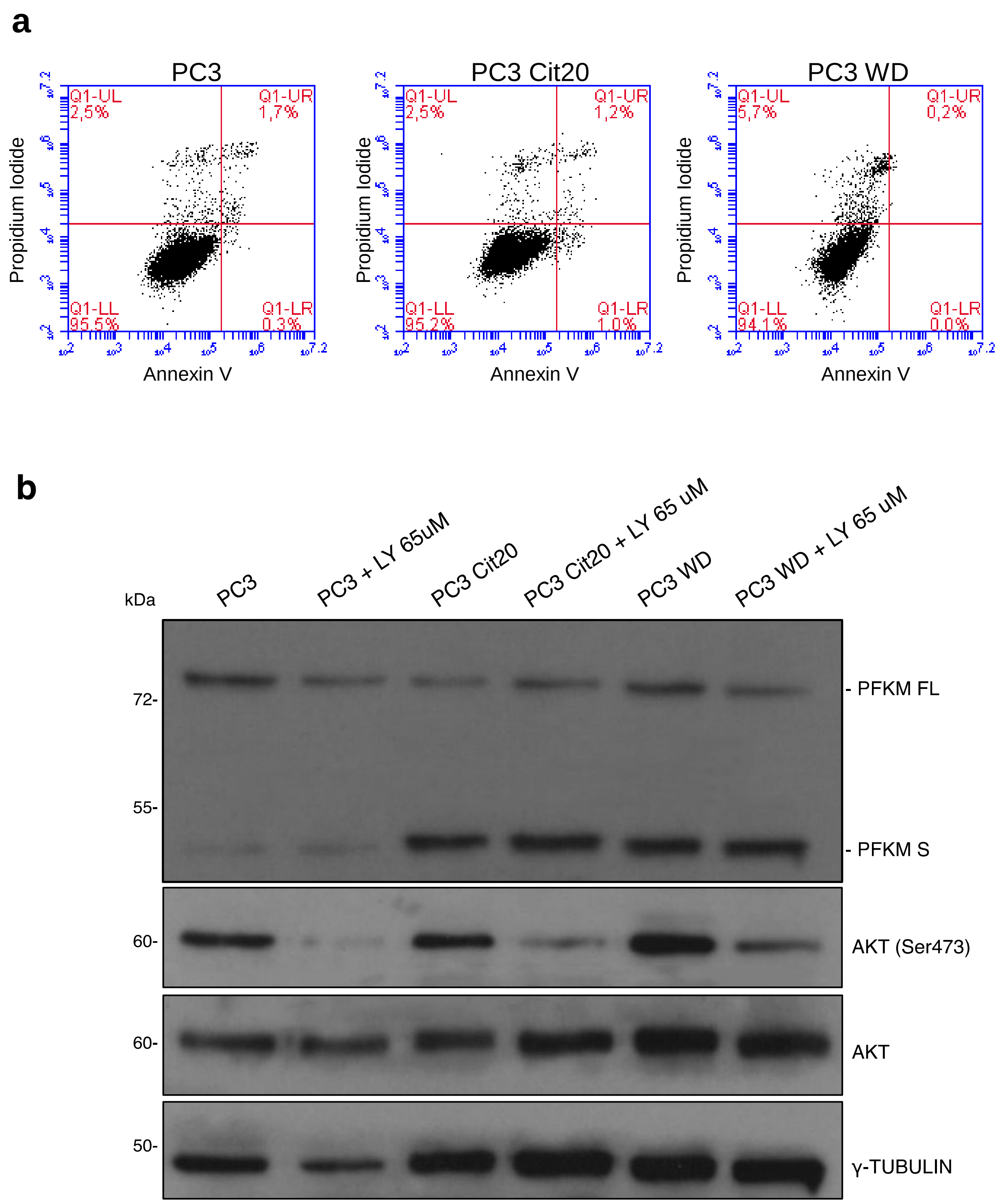

Figure S2. Citrate-resistant PC3 cells do not show sign of apoptosis.
(a) Flow cytometric detection of phosphatidylserine expression on PC3, PC3 Cit20 and PC3 Cit20 WD using fluorescein labelled Annexin V and Propidium iodide.

(b) **AKT inhibition does not affect the PFKM proteolysis.**
Lysates from PC3, PC3 Cit20 and PC3 Cit20 WD cells treated or not with the selective AKT inhibitor (LY294002) were analyzed by Western blot using the indicated antibodies. γ-tubulin was used as loading control.

Figure S3

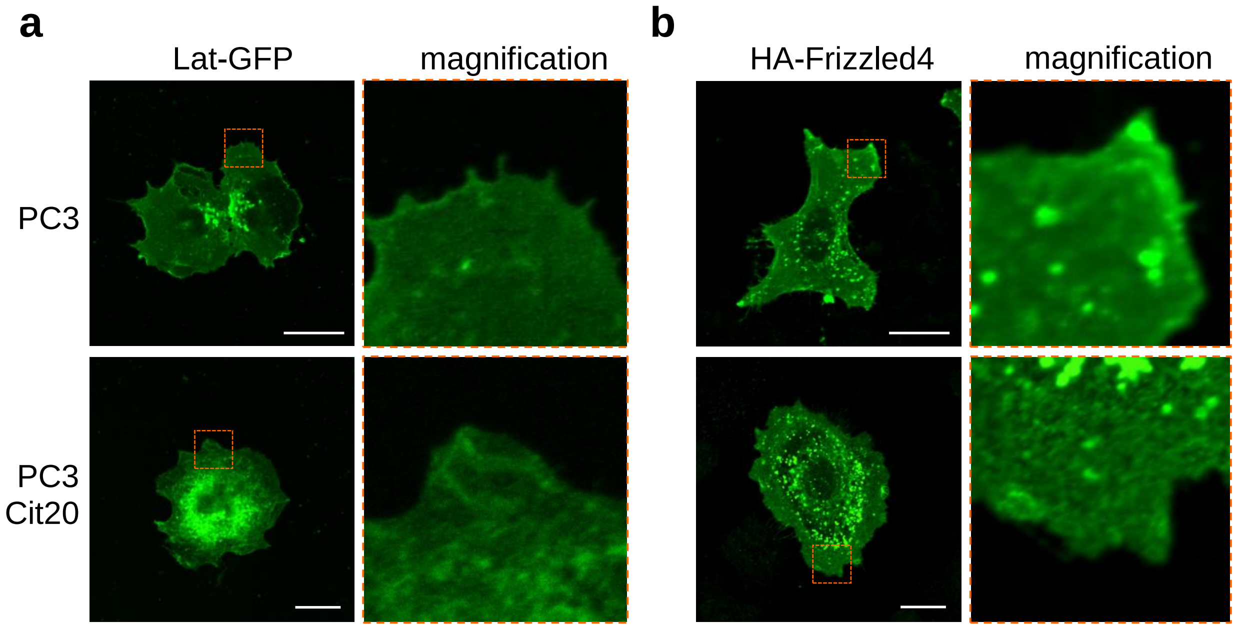

Figure S3. Analysis of plasma membrane-resident reporter proteins in PC3 Cit20 cells. PC3 and PC3 Cit20 cells were transiently transfected with the indicated reporter proteins (Lat-GFP and HA-Frizzled4). After 24 hours, cells were fixed and protein distributions were analyzed by confocal microscopy. HA-Frizzled4 was detected by using a mouse monoclonal antibody against HA-tag. Single focal sections are shown. Magnifications are shown at the right. Scale bar 10 µm.

Figure S4

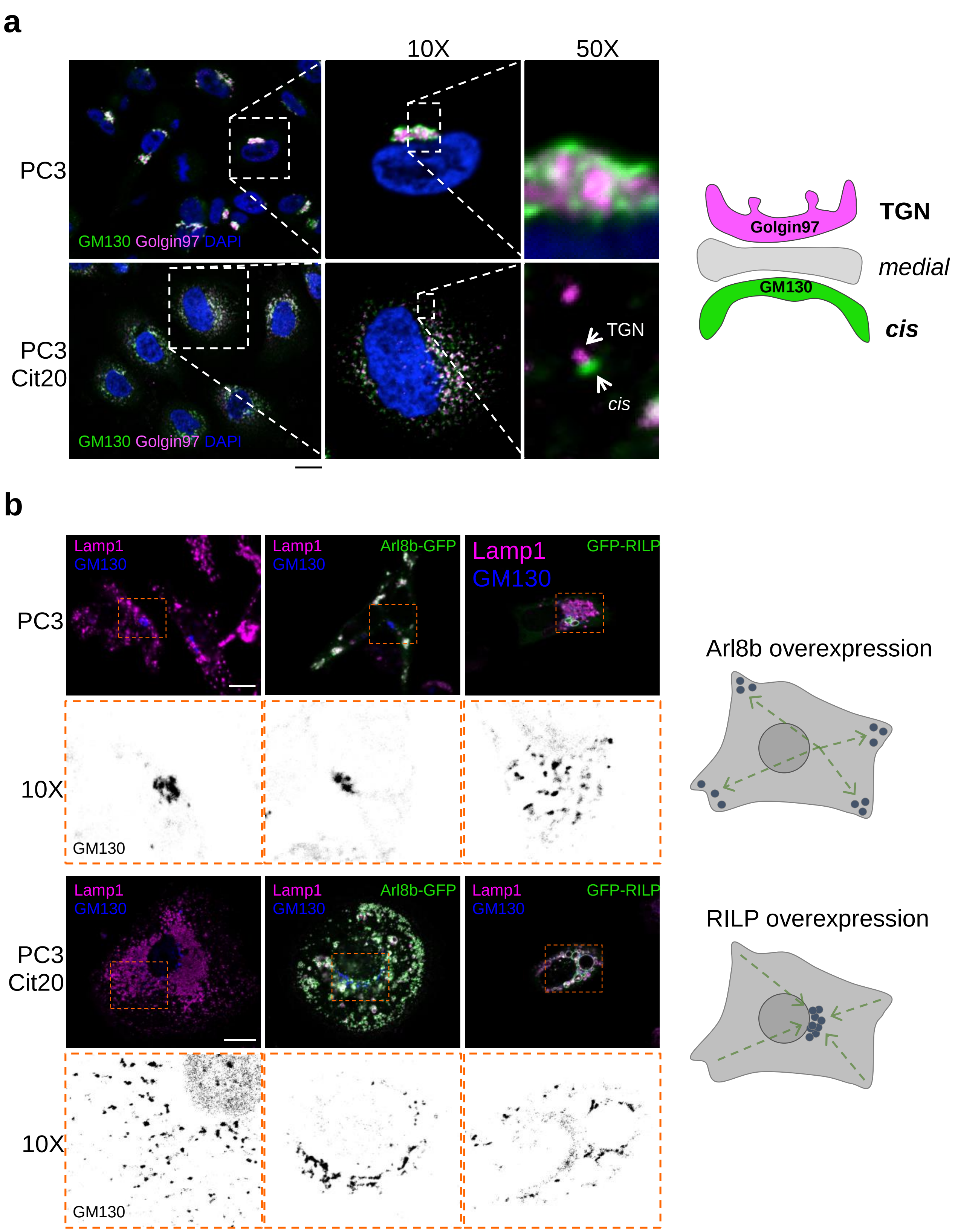

a
10X
50X
PC3
GM130 Golgin97 DAPI
PC3
Cit20
GM130 Golgin97 DAPI
TGN
cis
Golgin97
TGN
medial
GM130
cis
b
Lamp1
GM130
Lamp1
GM130
Arl8b-GFP
Lamp1
GM130
GFP-RILP
PC3
10X
GM130
Arl8b overexpression
Lamp1
GM130
Lamp1
GM130
Arl8b-GFP
Lamp1
GM130
GFP-RILP
PC3
Cit20
10X
GM130
RILP overexpression

Figure S4. Analysis of Golgi structure in PC3 Cit20 cells.

(**a)** Golgi morphology and structure were assessed by indirect immunofluorescence by using GM130 and Golgin-97, markers of cis- and trans-cisternae of Golgi complex, respectively. Magnifications are shown on the right. Arrows indicate the *cis*- and *trans*-Golgi network (TGN) labelled structures. Scale bar 10 μm. (**b**) PC3 and PC3 Cit20 cells were transiently transfected to exogenously over-express the indicated GFP-tagged proteins (Arl8 and RILP). Lysosomes morphology and positioning as well as Golgi structures were analyzed by indirect immunofluorescence by using the indicated antibodies (Lamp1 marker of lysosomes and GM130 marker of *cis*-Golgi). Single focal sections are shown. Magnifications are shown in black and white format below each respective original picture. Scale bar 10 μm.
